# Reliability and validity of the international physical activity questionnaire compared to calibrated accelerometer cut-off points in the quantification of sedentary behaviour and physical activity in older adults

**DOI:** 10.1371/journal.pone.0195712

**Published:** 2018-04-19

**Authors:** Declan J. Ryan, Jorgen A. Wullems, Georgina K. Stebbings, Christopher I. Morse, Claire E. Stewart, Gladys L. Onambele-Pearson

**Affiliations:** 1 Health Exercise & Active Living Research Centre, Department of Exercise & Sport Science, Manchester Metropolitan University, Cheshire, United Kingdom; 2 Research Institute for Sport and Exercise Sciences, Liverpool John Moores University, Liverpool, United Kingdom; Queen's University Belfast, UNITED KINGDOM

## Abstract

**Background:**

The aim was to determine the reliability and validity of IPAQ measured sedentary behaviour (SB) and moderate–vigorous physical activity (MVPA) in older persons whilst examining any sex differences in reliability and validity results.

**Method:**

89 participants (73.7 ± 6.3 years, 54% female) completed the IPAQ. Participants were fitted with a thigh mounted triaxial accelerometer (GeneActiv Original) for seven consecutive days and subsequently completed a second IPAQ.

**Results:**

IPAQ showed weak reliability qualities for Total SB (h·week^-1^) and 10 minute MVPA (accumulated in bouts ≥ 10 continuous minutes, h·week^-1^). IPAQ had poor concurrent validity qualities for Total SB, 10 minute MVPA, but not Sporadic MVPA (accumulated in bouts < 10 continuous minutes, h·week^-1^). IPAQ only categorised participant physical behaviour classification correctly 2% of the time. Sex differences were only present for the correlation slope of IPAQ 10 minute MVPA reliability measures.

**Conclusion:**

Our data suggests that the IPAQ is not suitable for assessing older adults habitual physical behaviour.

## Introduction

The International Physical Activity Questionnaire (IPAQ) is used worldwide to indirectly evaluate an individual’s volumes of sedentary behaviour (SB) and moderate to vigorous physical activity (MVPA) throughout the last seven-day week [1]. There is a suggestion that engaging in more than an hour a day of MVPA can attenuate the negative impact of prolonged SB on all-cause mortality [2]. However, given that older adults are expected to only spend 55% of their remaining years in ‘good’ health (at the age of 65 years) [3], there has been a focus on improving quality of life in remaining years rather than extending the amount of life years. Pre-clinical markers of cardiovascular disease (one of the most prevalent disease states in older adults [4]) have highlighted possible independent effects of SB and MVPA in our previous work [5, 6] and others [7–11]. Therefore, the ability to measure SB and MVPA separately is a purported strength of the IPAQ, particularly when attempting to establish links between SB, MVPA, and pre-clinical health status.

Sedentary behaviour is defined as any seated or reclined posture (e.g. sitting, lying down, and driving) that expends 1.50 or less, Metabolic Equivalent Tasks (METs) while MVPA is any activity that expends 3.00 or more METs [12]. One MET is equal to Resting Metabolic Rate (RMR) or 3.50 ml·kg·min^-1^ of oxygen utilisation, which is reported to reflect a 70 kg young person’s RMR [13].

MET thresholds are commonly applied to accelerometer data [14, 15] to allow for an objective measure of free-living SB and MVPA. Accelerometry is still a new concept within free-living monitoring, but is deemed a valid determination of SB and MVPA [16]. Nevertheless, in large epidemiological studies, the use of accelerometers can be costly and time consuming.

By contrast, self-report measures such as the IPAQ are readily available, very cost-effective and can generate large data sets. The IPAQ has previously undergone reliability and validity assessments in young to middle aged adults (*n* = 2721, 54% female, 36.8 ± 7.9 years) [1]. However, few studies have assessed its validity in populations over 60 years of age [17–19]. These aforementioned studies used populations that ranged from 54–325 participants (49% - 58% female) to validate the IPAQ against waist mounted accelerometer measures, which may not be as accurate as thigh mounted accelerometers when determining posture [20]. It is suggested that the IPAQ can provide reliable measures of SB and MVPA time in eastern older adult populations [17, 19], whose patterns of physical behaviour may not be representative of western older adult populations. Notwithstanding the above, the validity of the IPAQ is inconsistent in older adult populations [17, 19, 21, 22] with MVPA overestimations of 1090 mins∙week^-1^ and SB underestimations of 1646 mins∙week^-1^, compared to objective reports by Cerin, Barnett (19). Tomioka, Iwamoto (17) also found sex differences within IPAQ data sets. However, these differences were also present in hip-mounted accelerometer measures, which suggests that the IPAQ results were not due to sex differences in social desirability or approval [23]. To our knowledge no study, to date, has investigated the reliability and validity of the self-administered IPAQ Long-Form, English (last 7 days format) against thigh-mounted accelerometer derived data, using an older community-dwelling participant sample. Assessing the quality of the IPAQ across countries and age groups is essential to ensure the IPAQ is adaptable across different social, sex, and ethnic groupings.

Therefore, the objectives of this study were to compare two IPAQ Long-Form, English (last 7 days format) data sets, collected a week apart, for reliability assessment and to compare the results of the second completed IPAQ to accelerometer measures of free-living SB and MVPA for validity assessment. The aim was to provide a reliability and validity record for IPAQ measures of SB and MVPA in middle-class (e.g. coming from a sub-region characterized by proximity to several universities, low claimant unemployment rate, high proportion of managerial and professional posts), semi-rural UK-based older-adults. It was hypothesised that: 1. The IPAQ would be reliable. 2. Owing to a bias in the IPAQ to questions relating to PA rather than SB *per se*, the IPAQ may in fact underestimate SB and overestimate MVPA time. Thus, the second hypothesis was that the IPAQ may not be valid in quantifying SB and MVPA time. 3. Sex bias would not occur in both the reliability and the validity (i.e. accurate physical behaviour classification) of the IPAQ.

## Methods

Eighty-nine community-dwelling retired (and/or in voluntary employment) older participants (73.7 ± 6.3 years, 60–89 years, 54% female) who were independently mobile (i.e. no walking aids), did not suffer from an untreated cardiovascular disease, did not sustain an injury within the last three months, and were deemed generally healthy, volunteered to participate in the study. These participants were recruited primarily from older adult community groups within the Cheshire East Borough, England. The Manchester Metropolitan University: Exercise and Sports Science Ethics Sub-Committee granted ethical approval. Written informed consent was obtained prior to participation in the study. On recruitment, it was made clear to the participants that their inclusion within the study would require their physical activity/sedentary behaviours to remain habitual for at least the two weeks covered by the study protocol (see below).

During the first visit to the laboratory, 76 participants successfully completed the self-administered IPAQ Long-Form, English (last 7 days format), with the remaining participants returning incomplete questionnaires which were therefore not analysed. Following IPAQ completion, participants were then fitted with a commercially available thigh mounted (anterior aspect, at 50% of greater trochanter to femoral lateral condyle distance) triaxial accelerometer (GENEA, GENEActiv Original, Activinsights Ltd, Kimbolton, UK) on their dominant thigh, for a free-living week (7 consecutive days). Standing leg preference during a single leg balance exercise determined leg dominance. A waterproof adhesive patch (3M Tegaderm Film, North Ryde, Australia) was used to mount GENEA. GENEA recorded at a 60.0 Hz frequency and data were smoothed using 10 s epochs. The chosen GENEA output was Residual G (Residual G = √[[standard deviation x]^2^ + [standard deviation y]^2^ + [standard deviation z]^2^]), adapted from our previous work on total movement analysis in older persons (Onambele, Narici [24]) and termed the Cheshire Algorithm for Sedentarism (CAS). The SB (≤1.50 METs) cut-off point was 0.057 G and the MVPA (≥3.00 METs) cut-off point was 0.216 G. These cut-off points were derived from a systematic validation of the GENEA against expired gas samples of older adults collected during a laboratory-based activity calibration protocol, where ten ambulatory functions (i.e. 1-lying down, 2-sitting, 3-standing quietly, 4-self-selected ground walking, 5-brisk walk on treadmill, 6–3.5Km/hr walk on treadmill, 7-self-selected walk on treadmill, 8-self-selected weighted-vest treadmill walking (at 15% of body weight), 9-repeated side-stepping, 10-cycling) were monitored with concurrent gas analyses, heart rate, motion analysis and accelerometer output [5]. There was a strong explained variance between Residual G and METs (*r*^*2*^ = 0.89, *p* < 0.001). Residual G cut-off points and MET thresholds had a strong agreement in posture (Cohen’s kappa = 1.00, *p* < 0.001) and SB/PA intensity (Cohen’s kappa = 0.81, *p* < 0.001) identification [5]. During in house calibration, it was found that one MET was equal to the RMR of the participant to help account for individual differences in physical fitness. Following a week (uninterrupted 7 days of 24-hour-long accelerometer data collection) of GENEA wear-time (9 days in total, first and last day eliminated as the laboratory visits were on these days), 86 participants completed another self-administered IPAQ Long-Form, English (last 7 days format) within 8–11 days of the first laboratory visit. Scoring of the IPAQ was completed as described previously [25]. Due to older adults completing small amounts of MVPA [26], IPAQ measures of moderate physical activity (PA) and vigorous PA (including walking and cycling), accumulated in bouts of at least 10 minutes (a criterion within the IPAQ questions), were combined to make 10 minute MVPA (_10_MVPA) hours over a week (accumulated in ≥10 continuous minute bouts). To allow direct comparison with the IPAQ, Sporadic MVPA (sMVPA, accumulated in bouts <10 continuous minutes), as well as _10_MVPA hours over a week (accumulated in ≥10 continuous minute bouts), were used as the chosen output for GENEA. The 10 minute bout of MVPA criterion is included in the IPAQ and GENEA measures as this minimum bout length is required for MVPA bouts to contribute to the government recommended 2.5 h∙week of MVPA [27]. However, MVPA bouts of less than 10 minutes (sMVPA) have been suggested to also improve health status [28–31]. Therefore, sMVPA was included as a GENEA variable since the IPAQ may be able to provide a valid measure of sMVPA. Thus, the IPAQ could be useful for comparisons with health parameters. For SB, Total SB hours over a week was the chosen output of the IPAQ and GENEA, with the former including motorised transport and sitting time on a weekday and weekend. Participants who had less than seven days of valid GENEA data (three participants (1 male and 2 females) only had 6 valid days (i.e. full 24 hour-long data set), the mean value of each participant’s six valid days was added to the sum of their valid days to complete their week (7 day) data set. In some occurrences, the GENEA became detached whilst the participant was sleeping at night however, this was still considered a valid day if the GENEA was reattached when the participant woke up. As sleeping time was self-reported (naps not included), GENEA data was time stamped as Sleeping during these self-reported hours and therefore, Residual G had no effect on this classification. In light of the potential independence between SB and MVPA, it is now possible to be highly sedentary whilst also being physically active [32]. Therefore, physical behaviour levels were categorised into four groups [33–36], based on government MVPA recommendations [27] and evidence that suggests a maximum SB threshold of 8 h∙day^-1^ before an increased risk of all-cause mortality in older adults [37]: highly sedentary and physically inactive (couch potato), highly sedentary but physically active (active couch potato), low sedentary and physically inactive (ambulator), and finally, low sedentary and physically active (active ambulator). Even though validation of the IPAQ in non-English older adults has shown over and underestimation of MVPA and SB, respectively [19, 22, 38], the IPAQ may be sufficiently sensitive to classify physical behaviour phenotype, which have shown distinctions in health status [39]. Physical behaviour classification definitions are provided in Table 1. Only waking hours (with self-reported light-off time and wake-up time) are analysed for the purpose of this paper.

**Table 1:** Physical behaviour classification using mean SB per day and 10 minute MVPA per week. 10 minute MVPA threshold from National Health Service (27). SB threshold from Pavey, Peeters (37).

	SB ≥ 8 h·day^-1^	SB < 8 h·day^-1^
**10 minute MVPA < 2.5 h·week**^**-1**^	Couch Potato	Ambulator
**10 minute MVPA ≥ 2.5 h·week**^**-1**^	Active Couch Potato	Active Ambulator

## Statistical analyses

SPSS Statistics 21 (International Business Machines Corporation, New York, USA) was used for statistical analyses. Those who were missing first or second visit IPAQ data were removed from the relative and absolute between day IPAQ reliability assessments. Meanwhile individuals only missing first visit IPAQ data were included in the concurrent validity and physical behaviour classification agreement assessments against GENEA data. For hypothesis one, related to between day IPAQ reliability, with the data being non-parametric (based on the outcome of the Kolgomorov-Smirnov and Levene’s tests outcomes), Spearman rho correlations were used to examine the association between the first and second visit IPAQ data (for Total SB and _10_MVPA). To illustrate the absolute reliability between first visit and second visit IPAQ data sets, Bland-Altman plots were used.

For hypothesis two, related Samples t-tests (or Wilcoxon Signed Rank tests for non-parametric data sets) and Spearman rho correlations were used to assess the validity of the IPAQ against GENEA measures of Total SB, _10_MVPA, and sMVPA. To illustrate the concurrent validity of the IPAQ against GENEA, Bland-Altman plots were used.

For hypothesis three, independent samples t-tests (or Mann Whitney-U tests for non-parametric data sets) were used to examine sex differences in Total SB, _10_MVPA, and sMVPA. These sex differences were carried out for both IPAQ and GENEA data sets. To identify sex differences in correlation coefficients and correlation slopes for reliability and validity assessments, Fisher’s *z* transformation tests were performed. Differences in the residuals within the systematic bias between sexes were assessed with independent samples t-tests (or Mann Whitney-U for non-parametric data sets). Cohen’s Kappa test was used to assess physical behaviour classification agreement between the IPAQ and GENEA. Whilst Pearson’s Chi Squared was used to assess the effect of sex on physical behaviour classification.

Significance was set at *p* ≤ 0.05. Explained variance was expressed as weak (< 0.40), moderate (≥ 0.40, or strong (≥ 0.60). Data are presented as Mean ± Standard Deviation, or Median (Interquartile Range).

## Results

Sex differences were present for height and mass within the Completed (two IPAQ reports) and Missing first visit IPAQ (i.e. second visit IPAQ report only) groups (Table 2). Seventy-three participants (54% females) were included in the reliability assessment while 86 participants (52% females) were included in the validity assessment. Two females were then excluded from the validity assessment due to GENEA data errors.

**Table 2:** Demographics of participants that completed the IPAQ at both visits, or were missing either their first or second visit IPAQ data.

Group	Male (*n* = 41)	Female (*n* = 48)	Pooled (*n* = 89)
**Completed**	(*n* = 33)	(*n* = 40)	(*n* = 73)
**Age (years)**	74.6 ± 6.0	72.8 ± 6.8	73.7 ± 6.5
**Height (m)**	1.7 ± 0.1	1.6 ± 0.1^†^	1.6 ± 0.1
**Mass (kg)**	79.8 ± 11.0	71.4 ± 12.5*	75.2 ± 12.5
**BMI (kg·m**^**2**^**)**	27.6 ± 4.0	28.3 ± 5.0	28.0 ± 4.6
**Missing first visit IPAQ**	(*n* = 8)	(*n* = 5)	(*n* = 13)
**Age (years)**	76.1 ± 5.2	72.3 ± 5.7	74.3 ± 5.4
**Height (m)**	1.70 ± 0.1	1.6 ± 0.1*	1.7 ± 0.1
**Mass (kg)**	85.1 ± 8.9	70.0 ± 20.5	80.4 ± 15.6
**BMI (kg·m**^**2**^**)**	28.5 ± 3.7	27.2 ± 8.7	27.9 ± 5.8
**Missing second visit IPAQ**	(*n* = 0)	(*n* = 3)	(*n* = 3)
**Age (years)**		69.0 ± 6.1	69.0 ± 6.1
**Height (m)**		1.7 ± 0.1	1.7 ± 0.1
**Mass (kg)**		85.3 ± 14.1	85.3 ± 14.1
**BMI (kg·m**^**2**^**)**		31.1 ± 5.9	31.1 ± 5.9

* Parametric

^†^ Non-Parametric—Significantly different from male, *p* ≤ 0.05.

### Relative reliability of the IPAQ

**Fig 1:**
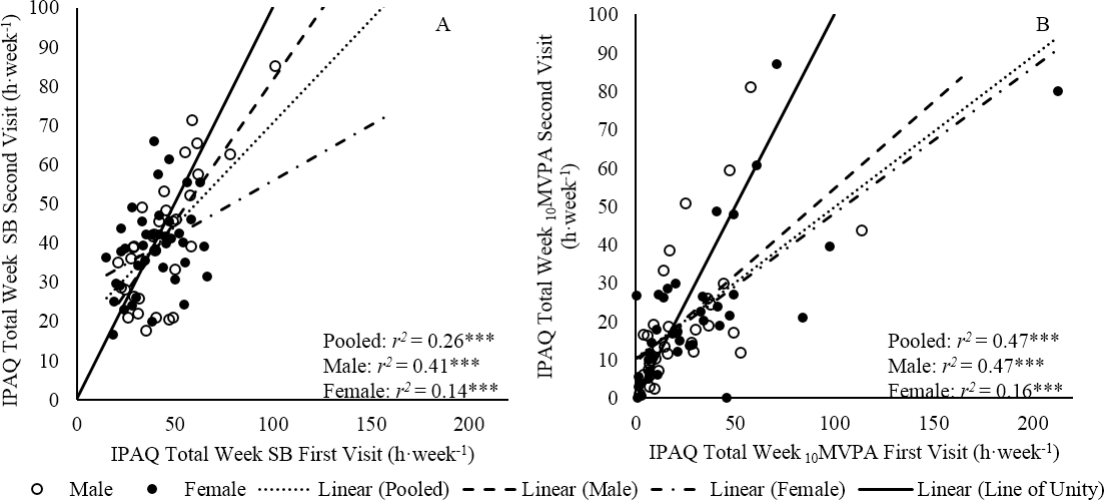
Reliability of the IPAQ expressed with Spearman rho. **(A)** Spearman rho correlation between first and second visit IPAQ Total SB. (B) Spearman rho correlation between first and second visit IPAQ _10_MVPA. * *p* ≤ 0.05, ** *p* ≤ 0.01, *** *p* ≤ 0.001. Note x-axis scale adjusted.

Within the pooled population, a weak correlation between first and second visit IPAQ data was present for Total SB (*r*^*2*^ = 0.26) (Fig 1A). For _10_MVPA, moderate correlations were found between first and second visit IPAQ data for the pooled population (*r*^*2*^ = 0.47) (Fig 1B). Interestingly, the majority of data points were situated below the line of unity for Total SB and _10_MVPA; suggesting that there was a trend for participants to report a lesser amount of time spent performing Total SB and _10_MVPA in the second visit IPAQ compared to the first visit IPAQ.

### Absolute reliability of the IPAQ

**Fig 2:**
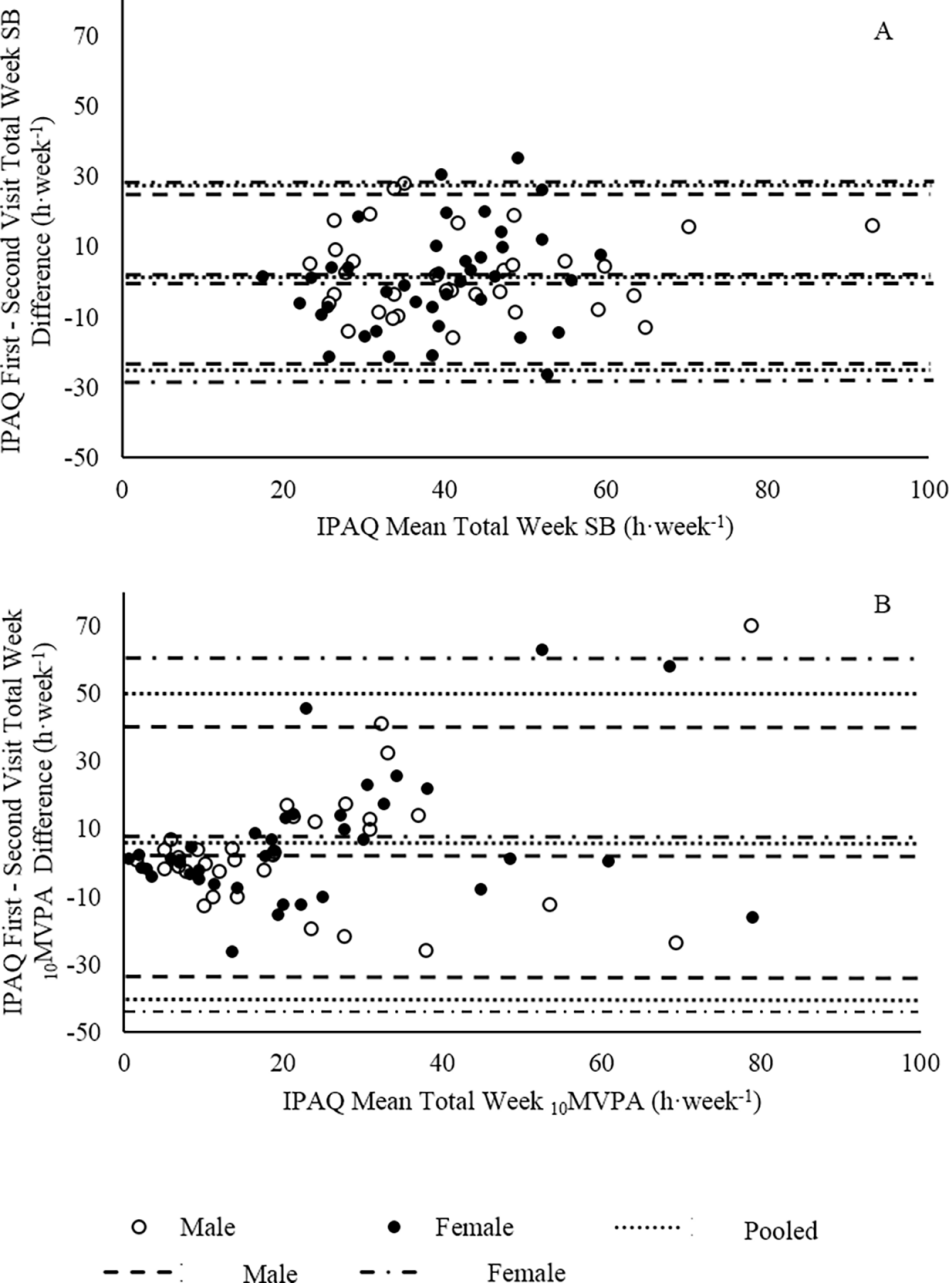
Reliability of the IPAQ expressed with Bland-Altman plots. (A) Bland-Altman plot of the difference between First and Second Visit IPAQ data against the Mean of the First and Second Visit IPAQ data for Total SB. (B) Bland-Altman plot of the difference between First and Second Visit IPAQ data against the Mean of the First and Second Visit IPAQ data for _10_MVPA. Lines represent systematic bias and 95% CI for pooled, male, and female populations.

A Bland-Altman plot (Fig 2A) of Total SB difference between first visit and second visit IPAQ for the pooled population showed small systematic bias (non-significant). However the 95% confidence intervals (95% CI) suggest large inter-individual random error (b = 1.51 ± 25.9 h·week^-1^). Systematic bias was similar as Total SB mean increased, suggesting no proportional bias (*p* = 0.21).

For _10_MVPA, a Bland-Altman plot (Fig 2B) of _10_MVPA difference between first visit and second visit IPAQ for the pooled population showed systematic bias (b = 6.37 ± 22.7 h·week^-1^, *p* = 0.025). It is notable that heteroscedasticity was present for the pooled population (Kolmogorov-Smirnov, *p* < 0.001). Thus, proportional bias was present due to the difference between first and second visit IPAQ _10_MVPA data increasing as the mean of first and second visit IPAQ _10_MVPA data increased (*p* < 0.001).

### Concurrent validity of the IPAQ

**Fig 3:**
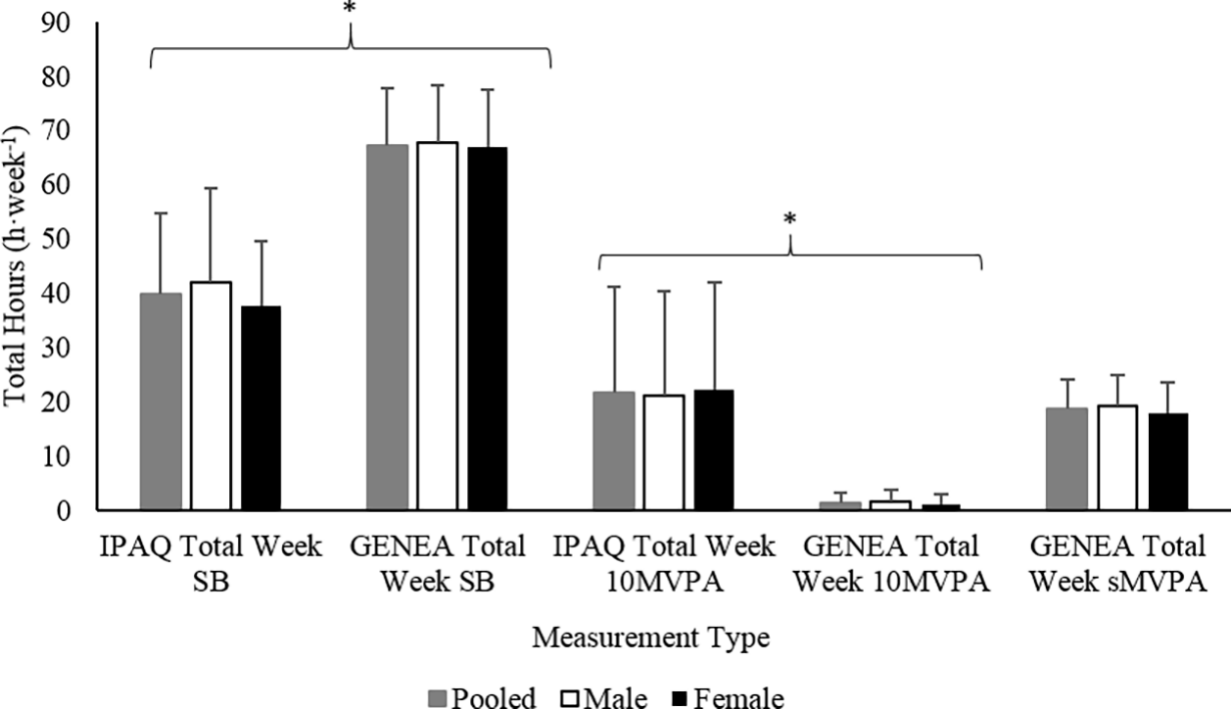
Comparison between IPAQ and GENEA measures of Total SB, 10 minute MVPA, and GENEA Sporadic MVPA. * *p* ≤ 0.05. Mean data presented, error bars represent standard deviation.

Total SB measured by the IPAQ (39.7 ± 14.9 h·week^-1^) was 41% (*p* ≤ 0.05) less than that of GENEA (67.3 ± 10.5 h·week^-1^) while _10_MVPA measured by the IPAQ (21.6 ± 19.4 h·week^-1^, Median 16.6 (8.63) h·week^-1^) was 15.7 fold (*p* ≤ 0.05) greater than that of GENEA (1.29 ± 1.95 h·week^-1^, Median 0.55 (0.17) h·week^-1^). Interestingly, IPAQ _10_MVPA was similar to GENEA sMVPA (21.6 ± 19.4 h·week^-1^, Median 16.6 (8.63) h·week^-1^, 18.5 ± 5.63 h·week^-1^, Median 18.3 (15.1) h·week^-1^, *p* > 0.05, respectively) (Fig 3).

**Fig 4:**
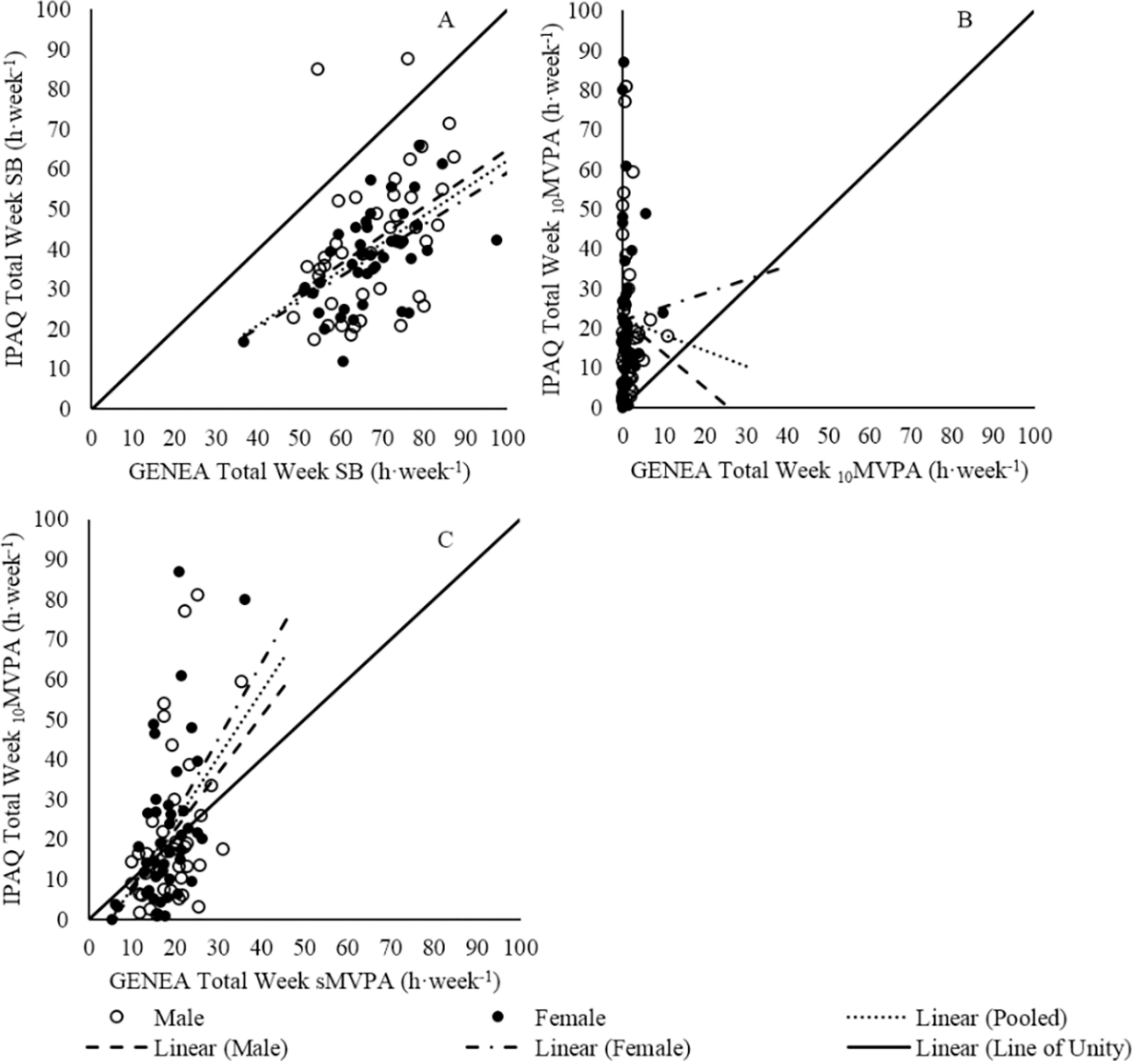
Validity between IPAQ and GENEA expressed with Spearman rho. (A) Spearman rho correlation between IPAQ and GENEA measures of Total SB. (B) Spearman rho correlation between IPAQ and GENEA measures of _10_MVPA. (C) Spearman rho correlation between IPAQ _10_MVPA and GENEA sMVPA. * *p* ≤ 0.05, ** *p* ≤ 0.01, *** *p* ≤ 0.001.

**Fig 5:**
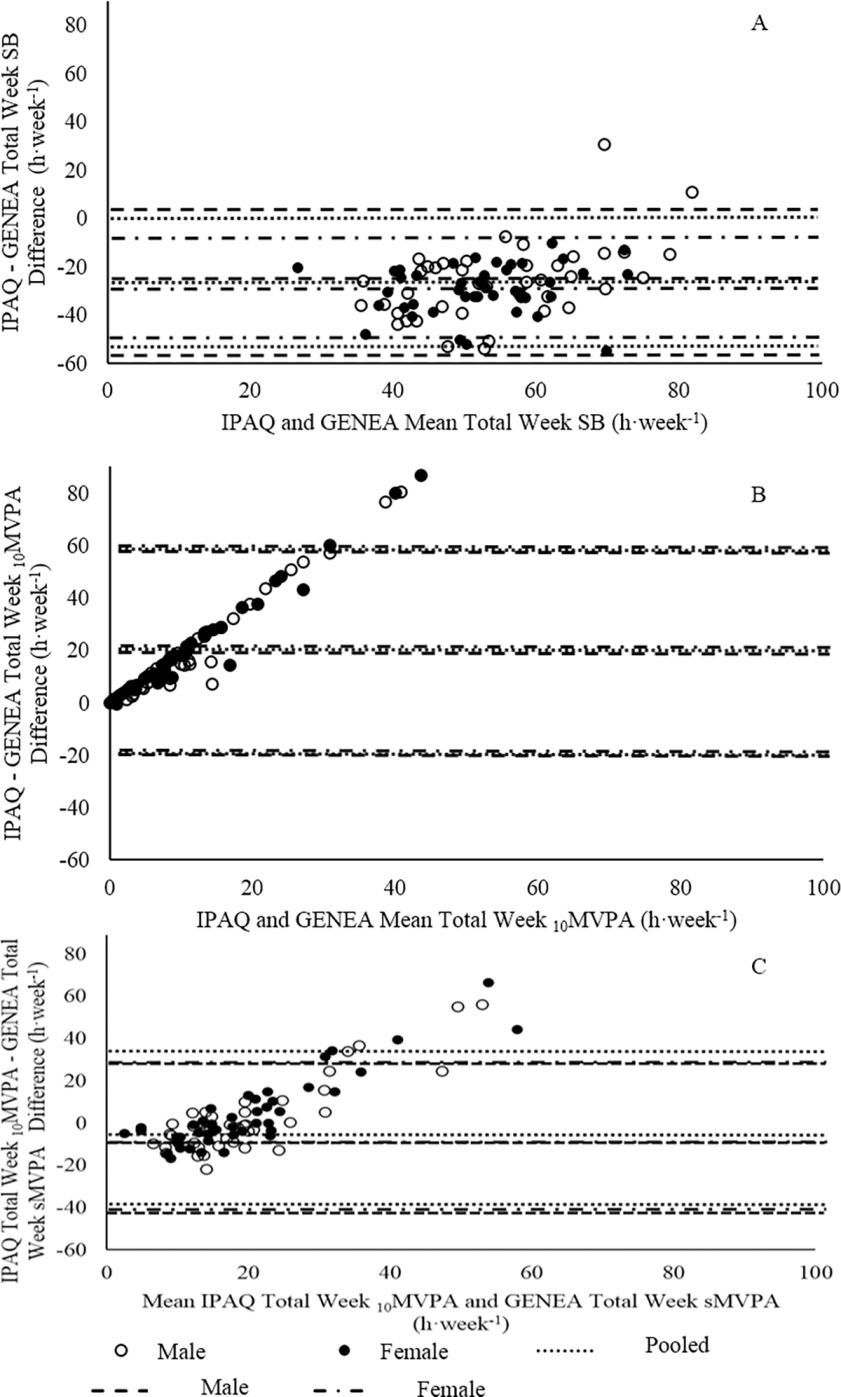
Validity between IPAQ and GENEA expressed with Bland-Altman plots. (A) Bland-Altman plot of IPAQ and GENEA Total SB. (B) Bland-Altman plot of IPAQ and GENEA _10_MVPA. (C) Bland-Altman plot of IPAQ _10_MVPA and GENEA sMVPA. Lines represent systematic bias and 95% CI for pooled, male, and female populations.

The pooled population showed a weak correlation between IPAQ and GENEA measures of Total SB (*r*^*2*^ = 0.29) (Fig 4A). Additionally, the majority of data points were situated below the line of unity; suggesting a trend for the IPAQ to under report Total SB when compared to GENEA measures. This is also supported by a Bland Altman (Fig 5A) plot that suggested all but two participants (11.4 h·week^-1^, 30.8 h·week^-1^) under-reported their Total SB using the IPAQ (pooled: b = -27.6 ± 26.5 h·week^-1^, *p* < 0.001). Proportional bias was present in the pooled population (*p* ≤ 0.001).

No significant correlation (*p* > 0.05) between IPAQ and GENEA measures of _10_MVPA was found for the pooled population (Fig 4B). Heteroscedasticity was present in _10_MVPA data sets (*p* ≤ 0.001). A Bland-Altman revealed systematic bias that suggested the IPAQ over reported _10_MVPA compared to GENEA (Fig 5B, b = 20.3 ± 19.6 h·week^-1^, *p* ≤ 0.001). The presence of proportional bias suggested the difference between IPAQ and GENEA measures of _10_MVPA increase as the mean of IPAQ and GENEA _10_MVPA increase (*p* ≤ 0.001).

A weak correlation between IPAQ _10_MVPA and GENEA sMVPA was present for pooled populations (*r*^*2*^ = 0.19) (Fig 4C). Heteroscedasticity was present for both _10_MVPAand sMVPA data sets (*p* ≤ 0.001). Interestingly, a Bland-Altman test suggested there was no systematic bias between IPAQ _10_MVPA and GENEA sMVPA measures (Fig 5C, b = 3.13 ± 17.5 h·week^-1^, *p* = 0.11). However, proportional bias was present (*p* ≤ 0.001).

### Sex differences

A moderate and weak correlation was found between first and second visit IPAQ Total SB data sets for males and females, respectively (Fig 1A). These correlations were not significantly different from one another (*z* = 0.62, *p* > 0.05). Additionally, male and female correlation slopes were similar (*z* = 1.48, *p* > 0.05). A Bland-Altman plot illustrated that the systematic bias between first and second visit IPAQ Total SB was non-significant for males and females (b = 2.91 ± 22.8 h·week^-1^, b = 0.61 ± 28.2 h·week^-1^, *p* > 0.05, respectively). Proportional bias was not present for either group (Fig 2A). There was no significant difference between the systematic bias of males and females (*p* = 0.53).

Moderate correlations between first and second visit IPAQ _10_MVPA data sets were found for males and females (Fig 1B). These correlations were not significantly different (*z* = 0.02, *p* > 0.05). However, male and female correlation slopes were significantly different (*z* = -2.20, *p* < 0.05). A Bland-Altman plot illustrated that the systematic bias between first and second visit IPAQ _10_MVPA was non-significant within males and females (Fig 2B, b = 3.64 ± 18.8 h·week^-1^, b = 8.62 ± 27.1 h·week^-1^, *p* > 0.05, respectively). The systematic bias was not different between males and females (*p* = 0.68). Proportional bias was present for females (*p* ≤ 0.001) but not for males (*p* = 0.08) (Fig 2B).

No sex differences were present within measures of Total SB, _10_MVPA, or sMVPA for second visit IPAQ and GENEA (Fig 3). Differences between IPAQ and GENEA measures of Total SB, _10_MVPA were found for males and females (*p* ≤ 0.05). No difference between IPAQ _10_MVPA and GENEA sMVPA was found for males and females (Fig 3).

Weak correlations were found between IPAQ and GENEA Total SB within males and females (Fig 4A), though there was no difference either between these correlations (*z* = -0.25, *p* > 0.05) or male and female correlation slopes (*z* = 0.22, *p* > 0.05). A Bland-Altman plot (Fig 5A) illustrated that there was systematic bias between IPAQ and GENEA Total SB for males and females (b = -25.9 ± 31.4 h·week^-1^, *p* < 0.05, b = -29.3 ± 20.7 h·week^-1^, *p* < 0.05, respectively). No difference in systematic bias was found between sexes (*p* = 0.25). Proportional bias was present for males (*p* ≤ 0.001) but not females (*p* = 0.32).

No correlation was found between IPAQ and GENEA _10_MVPA within males and females (Fig 4B). The correlations coefficients and slopes did not differ between sexes (*z* = -0.07, *p* > 0.05, *z* = -0.54, *p* > 0.05, respectively). A Bland-Altman plot illustrated that systematic bias was present for the difference between IPAQ and GENEA _10_MVPA within males and females (Fig 5B, b = 19.3 ± 19.8 h·week^-1^, *p* ≤ 0.001, b = 21.2 ± 19.6 h·week^-1^, *p* ≤ 0.001, respectively). Systematic bias was not different between sexes (*p* = 0.44). Proportional bias was present for both sexes (*p* ≤ 0.001).

Weak correlations between IPAQ _10_MVPA and GENEA sMVPA were present for males and females (*r*^*2*^ = 0.18, *p* ≤ 0.01, *r*^*2*^ = 0.22, *p* ≤ 0.01). These correlations were not different between sexes (*z* = -0.09, *p* > 0.05). Male and female correlation slopes were non-significantly different (*z* = -0.70, *p* > 0.05). A Bland-Altman plot (Fig 5C) illustrated no systematic bias between IPAQ _10_MVPA and GENEA sMVPA for males and females (b = 2.02 ± 17.7 h·week^-1^, *p* = 0.47, b = 4.17 ± 17.4 h·week^-1^, *p* = 0.12, respectively). Systematic bias was not different between sexes (*p* = 0.41). Proportional bias was present for both sexes (*p* ≤ 0.001).

The IPAQ and GENEA exhibited low agreement in the physical behaviour classification of the pooled (k = -0.01, *p* = 0.55), male (k = -0.03, *p* = 0.28), and female (k = 0.007, *p* = 0.69) populations (Table 3). Only 2% of physical behaviour levels were correctly classified by the IPAQ in the pooled, male, and female populations (Table 3). Sex was without effect on the ability for the IPAQ to correctly classify physical behaviour levels (Chi^2^ = 0.001, *p* = 0.97).

**Table 3:** Physical behaviour classification of the second IPAQ compared against that of GENEA.

IPAQ	GENEA				
	Couch Potato	Active Couch Potato	Ambulator	Active Ambulator	*IPAQ Total*
**Couch Potato**	NC	NC	NC	NC	*NC*
**Active Couch Potato**	9 (7 M, 2 F)	1 (0 M, 1 F)	NC	NC	*10 (7 M*, *3 F)*
**Ambulator**	5 (2 M, 3 F)	NC	NC	NC	*5 (2 M*, *3 F)*
**Active Ambulator**	47 (19 M, 28 F)	10 (7 M, 3 F)	11 (5 M, 6 F)	1 (1 M, 0 F)	*69 (32 M*, *37 F)*
***GENEA Total***	*61 (28 M*, *33 F)*	*11 (7 M*, *4 F)*	*11 (5 M*, *6 F)*	*1 (1 M*, *0 F)*	*84 (41 M*, *43 F)*

NC (not classified)–Based on their IPAQ data, participants were not classified into the group in question. M–males. F–females. *p* ≤ 0.05.

## Discussion

The objectives of this study was to compare two self-administered IPAQ Long-Form, English (last 7 days format) documents completed a week apart, for reliability assessment, by a middle-class older UK population and to evaluate the results of the second completed IPAQ to GENEA measures of free-living SB and MVPA, for validity assessment. The aims were to 1. Assess the reliability and validity of the IPAQ for SB and MVPA in these comparatively older-old, community-dwelling persons and 2. Determine any sex differences within the reliability and validity assessments. It was hypothesised that the IPAQ would provide a reliable (i.e. repeatable) measure of Total SB and _10_MVPA in relatively older persons but may not provide acceptable levels of external validity (i.e. when absolute data are compared against GENEA data sets). Additionally, it was thought that sex differences would not influence reliability and validity assessments of the IPAQ. The results of the current study suggest that one hypothesis can be upheld: the IPAQ, show low reliability/repeatability qualities, and does not provide acceptable levels of validity when compared to GENEA measures of Total SB and _10_MVPA in older persons. As expected, sex differences did not influence reliability and validity assessments of the IPAQ.

### Reliability of the IPAQ

Relative reliability of the IPAQ exhibited a significant repeated measures correlation coefficient (*r* = 0.59; *r*^*2*^ = 0.26, *p* < 0.001) for Total SB. This is lower than that reported in the relatively younger population (36.8 ± 7.93 years) used in the original 12-country IPAQ study [1] (*r* = 0.83 ± 0.06, *p* ≤ 0.05) and lower than the UK population (*r* = 0.84, *p* ≤ 0.05) used in the aforementioned study. Whether the geographical location and therefore socio-economic factors of the participants (current study: Cheshire East Borough, vs. Craig, Marshall (1): Bristol) played a role in the differences between studies is unknown. It could be argued that, unlike the participants in our current study, the adults in the study of Craig, Marshall (1) were still employed and therefore, had a predictable structure in their day-to-day lives. However, older adults have been shown to also have similarly predictable daily routines [40], which they highly value [41]. Furthermore, the lack of systematic bias (Fig 2a) in the difference between first and second visit IPAQ Total SB may be highlighting this predictability in daily routines (Monk, Reynolds (40). On the other hand, large 95% CI (b = 1.51 ± 25.9 h·week^-1^) question the reliability of the IPAQ.

The IPAQ measure of _10_MVPA also displayed a moderate relative reliability coefficient (*r* = 0.69, *r*^*2*^ = 0.47, *p* < 0.001) but showed systematic bias (b = 6.37 ± 22.7 h·week^-1^, *p* = 0.025), thereby putting in question the reliability of the IPAQ for quantifying absolute MVPA in older persons. Unfortunately, our data cannot be compared with the 12-country IPAQ study as the previous study did not provide a suitable comparable measure of MVPA [1]. However, the results of the current study are similar to previous IPAQ–Long Form reliability measures in Belgian older adults (*r* = 0.63) [22] and outperformed the reliability of the IPAQ–Long Form, Chinese (7 day format) which found no significant between week correlation [19]. The presence of positive systematic bias (Fig 2B) and the majority of data points below the line of unity for _10_MVPA (Fig 1B) may illustrate a learning effect following the completion of the first visit IPAQ or greater awareness of _10_MVPA engagement. The greater awareness may be a result of a weeklong lifestyle surveillance by GENEA. However, is it hoped that the discrete and unrestrictive placement of GENEA (mid-thigh) would minimise any effect on the participant’s awareness of lifestyle.

### Validity of the IPAQ

For measurement of time spent performing SB or MVPA, accelerometry is considered the gold standard [42]. Moreover thigh-mounted accelerometry is deemed the most suitable for SB measures due to the change in thigh orientation that is common to the transition from standing to seated or reclined postures [43]. A strength of the current study is that it used a thigh-mounted triaxial GENEA that records the amount of static acceleration due to gravity. Using this knowledge, the in-house developed algorithm (CAS [44]) can calculate thigh orientation relative to the Earth’s surface to accurately determine whether the participant is standing, sitting down, lying sideways, or prone. Furthermore, unlike earlier research that applied accelerometer cut-points, which were not validated for the population being studied, the CAS uses cut-off points validated against the energy expenditure (METs) of aged-matched older adults (in our laboratories) to determine the time spent performing different PA intensities.

Participants had great difficulty in reporting Total SB using the IPAQ and often spent a few minutes formulating an answer. Although, significant correlations between IPAQ and GENEA for Total SB were present (Fig 4A), differences in average time (Fig 3) and systematic bias (27.6 ± 26.5 h·week^-^1, *p* < 0.001) revealed that the IPAQ underestimated Total SB by 41%. Only two participants over-reported their amount of Total SB (+11.4 and +30.8 h·week^-1^). This underestimation is consistent with another IPAQ validity study (hip mounted accelerometer) (b = 27.4 ± 3.46 h·week^-1^, *p* ≤ 0.001) that used older adults (*n* = 94, 65–85 years) [19]. Under-reporting is a common problem, qualitative research has shown that the sitting questions create confusion, as *“sitting on a weekday*?*”* does not provide details on which ‘day’ to report [45, 46]. Sedentary behaviour is viewed as a negative lifestyle choice by older adults [47]. Therefore, participants may have reported the day that had the least amount of SB to appear socially desirable. This is supported by the negative association found between social desirability and self-reported SB in other populations [48]. Alternatively, this difficulty in recall may also be due to the sporadic nature of SB and, unlike MVPA, engagement in other behaviours that occur at the same time as SB (e.g. eating, reading, and driving). Qualitative data was not the focus of this study; however, participant’s recollection of SB often came in the form of TV viewing. Therefore, SB such as eating may have been overlooked.

For _10_MVPA, a large systematic bias was also present as participant‘s IPAQ data over-reported by 20.3 ± 19.8 h·week^-1^ (*p* < 0.001) equivalent to, on average, 15.7 fold greater than total weekly _10_MVPA time. This overestimation is consistent with previous IPAQ research that used accelerometry (hip mounted) in older adults [19, 22, 38]. This, like SB, may be a result of social desirability as older adults view PA as a positive lifestyle choice [41]. Alternatively, participants may be reporting MVPA bouts that were less than 10 continuous minutes in duration. The results of the current study revealed weak correlations between IPAQ _10_MVPA and GENEA sMVPA (Fig 4C, *r*^*2*^ = 0.19, *p* ≤ 0.001). However, there was a small non-significant systematic bias between the two aforementioned variables (Fig 5C, b = 3.12 ± 17.5, *p* = 0.106) and no statistical difference between the population means of these two variables (Fig 3). Heesch, Van Uffelen (46) suggested that the structure of the questions may be leading to this problem as the, "*report activities lasting ≥ 10 minutes per session"* instruction is included in the `number of days the activity was performedˊ question and not the following question relating to the duration of one of those activities. This could be interpreted that the 10-minute criterion only relates to the frequency of the activity per week and not the duration of that activity.

### Sex differences

There was only one incident of a sex difference in the reliability of PA measures. For the relative reliability of _10_MVPA, males correlation slope was found to be greater than females (Fig 1B, *z* = -2.20, *p* < 0.05). Males and females had similar levels of GENEA measured _10_MVPA, sMVPA, and Total SB (Fig 3) therefore, it seems unlikely that one of the groups performed tasks that were not as memorable during the monitoring week [49]. Unfortunately, participants did not wear GENEA prior to the completion of the first visit IPAQ. Therefore, we cannot be sure whether this suggested sex difference was a result of changeable memory within groups or actual inter-week behaviour change [42].

As a result of the IPAQ overestimating _10_MVPA and underestimating Total SB, the majority of participants were placed into a higher physical behaviour classification than they actually were (Table 3). A previous attempt to correctly classify older adults (66–85 years) as physically active had found an 81% (k = 0.448, *p* < 0.001) agreement between IPAQ and waist mounted accelerometer data [18]. For direct comparison, of the 12 participants that were physically active in the current study, based on GENEA data (Active Couch Potato and Active Ambulator), 100% of those participants were correctly classified as physically active by the IPAQ. However, an additional 62 participants were also classed as physically active by the IPAQ causing only 14% of participants to be classified as physically inactive by the IPAQ. Overall, the IPAQ was only able to correctly classify 2% (k = -0.01, *p* = 0.55) of the participants into one of the four physical behaviour groups for the pooled, male and female populations, causing no participants to be classed as a Couch Potato (Table 3). In addition, sex had no effect on the ability of the IPAQ to correctly classify physical behaviour levels (Chi^2^ = 0.001, *p* = 0.97).

### Study limitation

The age of the participants in our current study ranged from 60–89 years, which only has a 9 year overlap with population for whom the IPAQ was originally validated for (15–69 years) [1]. Arguably, the reduced reliability of the IPAQ, could be partially assigned to this fact. Notably however previous studies have not made this distinction and have indiscriminately used the IPAQ in similarly much older population [50] and yet have not reported any necessity to improve the uptake of the questionnaire through population stratification by decade of age, thereby supporting our current design that includes persons aged 60 through to 89 years. A further limitation may be the use of accelerometers to measure physical behaviour as there are some activities that accelerometers do not capture adequately [51]. However, without the ability to visually observe (e.g. video monitor) and directly measure energy expenditure (e.g. using doubly labelled water) in free-living conditions, accelerometry, in particular thigh mounted, would arguably be the most suitable option for an objective measure of physical behaviour.

## Conclusion

This is one of the first studies to use thigh-mounted accelerometry (considered the gold standard for SB and MVPA measurement) as a validator for the self-administered IPAQ–Long Form, English (last 7 days format) in older adults. The results of the current study suggest that use of the self-administered IPAQ–Long Form, English (last 7 days format) in the ‘older-old’ adults shows little relative and absolute reliability when re-tested in an 8–11 day window, even after accounting for the fact that there two weeks of monitoring where self-reporting as ‘typical’. A weak correlation found between the IPAQ and GENEA measures of Total SB, is supported by a Bland-Altman plot that revealed Total SB is underestimated by the IPAQ. This suggests that the IPAQ may not be suitable for quantitatively measuring SB in older adults. Similarly, the IPAQ overestimated the amount of _10_MVPA and this was likely due to participants reporting MVPA bouts that were less than 10 continuous minutes, as there was no difference or systematic bias between IPAQ _10_MVPA and GENEA sMVPA measures. Therefore, the IPAQ may not be suitable for measuring MVPA, which consists of bouts at least 10 continuous minutes in duration. Due to underestimation of SB and overestimation of _10_MVPA by the IPAQ, only 2% of participants were correctly categorised into one of the four physical behaviour groups. Interestingly, no difference in reliability or validity measures were found between sexes apart from correlation slope measures of _10_MVPA reliability. Based on these results, it is suggested that the IPAQ should not be used as a monitoring technique to qualitatively classify or quantitatively measure habitual physical behaviour in older adults. Research aiming to monitor SB and PA should use an objective measurement technique where possible. The objective of future research should be to increase the number and precision of the questions in the self-administered IPAQ Long-Form, specifically addressing SB, English (last 7 days format) to make it more relevant to older adults (e.g. changing examples of activity), with the aim to improve its reliability and validity.
